# Resistance of Superhydrophobic Surface-Functionalized TiO_2_ Nanotubes to Corrosion and Intense Cavitation

**DOI:** 10.3390/nano8100783

**Published:** 2018-10-02

**Authors:** Weidi Hua, Piyush Kar, Partha Roy, Lintong Bu, Lian C. T. Shoute, Pawan Kumar, Karthik Shankar

**Affiliations:** 1 Department of Electrical and Computer Engineering, University of Alberta, 9211-116 St, Edmonton, AB T6G 1H9, Canada; weidi@ualberta.ca (W.H.); pkar1@ualberta.ca (P.K.); partharoy@curaj.ac.in (P.R.); lbu@ualberta.ca (L.B.); lshoute@ualberta.ca (L.C.T.S.); pawan@ualberta.ca (P.K.); 2 Department of Chemistry, Central University of Rajasthan, NH-8, Bandar Sindri, Rajasthan 305817, India

**Keywords:** superhydrophobic, resilient, cavitation, self-assembled monolayer, contact angle

## Abstract

The availability of robust superhydrophobic materials with the ability to withstand harsh environments are in high demand for many applications. In this study, we have presented a simple method to fabricate superhydrophobic materials from TiO_2_ nanotube arrays (TNTAs) and investigated the resilience of the materials when they are subjected to harsh conditions such as intense cavitation upon ultrasonication, corrosion in saline water, water-jet impact, and abrasion. The TNTAs were prepared by anodization of Ti foil in buffered aqueous electrolyte containing fluoride ions. The hydrophilic TNTAs were functionalized with octadecylphosphonic acid (ODPA) or 1H, 1H′, 2H, 2H′-perfluorodecyl phosphonic acid (PFDPA) to form a self-assembled monolayer on the TNTA surface to produce superhydrophobic ODPA@TNTA or PFDPA@TNTA surfaces. The superhydrophobic ODPA@TNTA and PFDPA@TNTA have contact angles of 156.0° ± 1.5° and 168° ± 1.5°, and contact angle hysteresis of 3.0° and 0.8°, respectively. The superhydrophobic ODPA@TNTA and PFDPA@TNTA were subjected to ultrasonication, corrosion in saline water, and water-jet impact and abrasion, and the resilience of the systems was characterized by electrochemical impedance spectroscopy (EIS), contact angle (CA) measurements, diffuse reflectance Fourier transform infrared spectroscopy (DRIFTS), and field-emission scanning electron microscopy (FESEM). The results presented here show that superhydrophobic ODPA@TNTA and PFDPA@TNTA are robust and resilient under the harsh conditions studied in this work, and indicate the potential of these materials to be deployed in practical applications.

## 1. Introduction

The production of liquid-repellent surfaces is an important and popular research topic. Owing to a wide variety of applications of anti-wetting surfaces such as anti-icing surfaces, anti-fouling surfaces, drag reduction, enhanced heat transfer, protein adsorption and liquid separations, interest in these fields have grown exponentially over the last few decades [1,2,3,4,5]. 

A surface is deemed to be superhydrophobic when the contact angle (CA) of water on the solid surface is 150° or greater [6,7]. The CA values of liquid droplets on a microscopically rough surface can be described theoretically by the Wenzel and Cassie-Baxter models [8]. According to the Wenzel model, the interaction of a liquid with a rough substrate results in the stabilization of liquid droplets through an increase in the contact area of the liquid, as it can wet the grooves of the rough substrate, while in the Cassie-Baxter model, the assumption is that the contact area between liquid and rough substrate is minimal because of the trapped air in the grooves of the rough micro- and/or nanoscale surfaces. 

In most applications that employ superhydrophobic coatings, a stable Cassie-Baxter state is more desirable than a Wenzel state, because in the Wenzel state the water droplet has a high tendency to adhere to the surface, thereby undermining the self-cleaning, antifouling, and other desirable properties of the materials [8,9]. Therefore, many different techniques have been employed to fabricate rationally designed rough textures on surfaces in order to minimize the liquid–solid contact area fraction parameter and enhance the generation of Cassie-Baxter states [10,11,12,13,14]. As a result, through both experiments and theoretical deductions, a microscale/nanoscale dual or hierarchical structure is considered an ideal texture for a superhydrophobic surface [15,16,17,18,19]. However, little attention has been paid to the durability of superhydrophobic surfaces against harsh conditions. In fact, the durability of superhydrophobic surfaces has been termed as the single biggest obstacle preventing their deployment in real world applications [20].

An elegant method to improve CA and impart chemical and mechanical stability, as well as surface durability, is the formation of a self-assembled monolayer (SAM) on nanostructured ceramic surfaces [21,22,23,24]. The ceramic (typically a metal oxide) provides chemical resilience and wear resistance, while the organic monolayer contributes to the low-energy, water-repellent properties of the surface. Alkylphosphonic acid and arylphosphonic acid SAMs on metal oxide surfaces are used for various applications, e.g., sensors, surface passivation, and crystal design [25,26,27,28]. These SAMs form a strong covalent bond with metal oxides resulting in ordered, closely packed monolayers with a high packing density of alkyl groups that are chemically stable and impart a high degree of hydrophobicity to the surface [29]. Some notable reports in this realm include stabilization of a superhydrophobic polybenzoxazine surface over a wide pH range [30], superhydrophobic aluminum and its alloys by their immersion in acidic and basic solutions for elongated periods [31], coating of Al_2_O_3_ with perfluorodecyltrichlorosilane for mechanical durability and improved corrosion resistance [32], the creation of surface superhydrophobicity for a variety of environmental conditions [33,34,35,36], and pentafluorophenyl surface functionalization of fuel cell carbon supports for increased corrosion resistance [37,38]. Titania is a ceramic material with facile fabrication techniques that forms nanostructures with different morphologies that are suitable for obtaining superhydrophobic surfaces. Self-assembled monolayer-coated TiO_2_ nanotube arrays are ideal systems with which to study the Cassie-Baxter wetting state [39], as they are known to be highly chemically and mechanically stable [40,41]. It is important to note that surface-functionalized TiO_2_ nanotube arrays have been used in a whole host of device applications across multiple disciplines such as label-free interferometric biosensors [42], stem cell differentiators [43], cell adhesion modifiers [44], dental implants [45], solar cells [46], and photocatalysts [47], due to which the study of the dynamic wetting properties and the resilience of the self-assembled monolayer-coated TiO_2_ nanotubes assumes special significance. 

In this work, we reported on the preparation, characterization, and resilience test of superhydrophobic TiO_2_ nanotube arrays (TNTAs) obtained by coating a conformal SAM using a simple method that involved immersing TNTA in alkyl or perfluoroalkyl phosphonic acid solution. Two different types of SAMs were used for the coatings—octadecylphosphonic acid (ODPA) and 1H, 1H′, 2H, 2H′-perfluorodecyl phosphonic acid (PFDPA). To test the resilience of the as-prepared materials, a high-power ultrasonic wave was applied to the SAM coated superhydrophobic TNTA, thereby subjecting the surface structure to cavitation and elastic vibrations of the surrounding medium. The resilience of the mechanical and chemical properties of the SAM-functionalized TNTAs against ultrasonic cavitation, corrosion in saline water, water-jet impact, and abrasion was characterized using diffuse reflectance infrared Fourier transform spectroscopy (DRIFTS), CA measurements, field-emission scanning electron microscopy (FESEM), and electrochemical impedance spectroscopy (EIS). 

## 2. Materials and Methods

### 2.1. Materials

Ti foil (99%, 0.89 mm thickness) and octadecylphosphonic acid, C_18_H_37_PO_3_H_2_ (ODPA) (97%), were obtained from Alfa Aesar. Sodium fluoride (≥99%), sodium chloride (≥99%), acetone (99.5%), methanol (99.8%), and citric acid monohydrate (HO_2_CCH_2_)_2_C(OH)(CO_2_H)·H_2_O (99%) were purchased from Fisher Scientific. 1H, 1H′, 2H, 2H′-perfluorodecyl phosphonic acid, CF_3_(CF_2_)_9_(CH_2_)_2_PO_3_H_2_ (PFDPA) was purchased from Aculon Inc (Aculon Inc. Sorrento Valley, San Diego, CA, USA). These chemicals were used as received without further purification. De-ionised (DI) water was used throughout this study, and all other solvents used were of HPLC grade.

### 2.2. Synthesis of TiO_2_ Nanotubes 

Titanium dioxide nanotube arrays (TNTAs) were prepared by electrochemical anodization at room temperature using a two-electrode setup. The obtained Ti foil was cut into 1 cm × 4 cm pieces and degreased by sonication in water, methanol, and acetone each for 10 min to remove contaminants before being used as the anode. Another piece of Ti foil with a surface area of 1 cm × 2 cm was used as the cathode and immersed in the electrolyte with the help of an O ring while maintaining a 5 cm inter-electrode distance. A Direct current (DC) power supply (9312-PS Variable Bench-top Power Supply, MPJA Inc, West Palm Beach, FL, USA) was employed to drive the anodic synthesis of TNTAs. The electrolyte solution consisted of 0.1 M citric acid and 0.1 M sodium fluoride dissolved in DI water. The pH of the solution was maintained at 5 by adding an adequate amount of NaOH solution. Thereafter, the Ti foil was anodized at 25 V for 15–17 h. The obtained TNTAs were sonicated for 5–10 min and then washed in methanol to remove debris layer and residual electrolyte.

### 2.3. Surface Functionalization

The freshly prepared TNTAs were surface-functionalized with octadecylphosphonic acid (ODPA) and perfluorodecylphosphonic acid (PFDPA) SAMs. To achieve a conformal surface coating of TNTAs with ODPA and PFDPA monolayers, the as-prepared TNTAs were immersed in 1 mM octadecylphosphonic acid (ODPA) and perfluorodecylphosphonic acid (PFDPA) solutions in methanol, respectively, at room temperature for 18–20 h. Subsequently, the surface modified TNTAs were rinsed with methanol to remove any physisorbed molecules and then dried thoroughly with gentle N_2_ gas flow. To compare the performance of SAMs and paint coating of TNTA, a RUST-OLEUM^@^ (Rust-Oluem Corporation, Concord, ON, Canada) commercial coating was used. The commercial coating was applied on the TNTA surface by spraying followed by drying for 12 hours. 

### 2.4. Characterization and Testing 

The morphological features of the TNTAs were determined using field emission scanning electron microscopy (FESEM) on a Zeiss Sigma FESEM equipped with GEMINI in-lens detector at an acceleration voltage of 5 keV. Ultrasonication was performed using a horn-sonicator operated at an output power of 225 W. To monitor the change in surface groups and to verify the surface functionalization of TNTAs, DRIFTS spectra of samples were recorded by mixing scraped-off functionalized TiO_2_ nanotube powder with Fourier transform infrared spectroscopy (FTIR) grade KBr and pressed to form a pellet in a sample cell. DRIFTS spectra were collected using a iS5 FTIR spectrometer (Thermo Nicolet Nexus670, Thermo Nicolet, Bunker Lake Boulevard Ramsey, MN, USA) equipped with a DRIFTS accessory. Drop Shape Analyzer (DSA) 100 (Krüss GmbH, Hamburg, Germany) was used for the static CA and CA hysteresis measurements by the pendant drop technique (4 µL drop volume) and monitoring with an in-built Charge-coupled device (CCD) camera. CA hysteresis was obtained from the difference in the advancing and receding CAs determined from the water droplet volume expansion and contraction, respectively, achieved by adding and withdrawing a water droplet placed on the surfaces of the ODPA@TNTA and PFDPA@TNTA. At least 3–5 different sample locations were chosen to determine the average static CA and CA hysteresis. Electrochemical experiments that included EIS were performed using a three-electrode electrochemical cell with Ag/AgCl reference electrode and a CHI660E potentiostat (CH Instruments, Inc., Austin, TX, USA). The electrolyte used was 3.5 wt. % NaCl aqueous solution.

## 3. Results and Discussion

### 3.1. Morphological Characterization of TNTAs 

**Figure 1:**
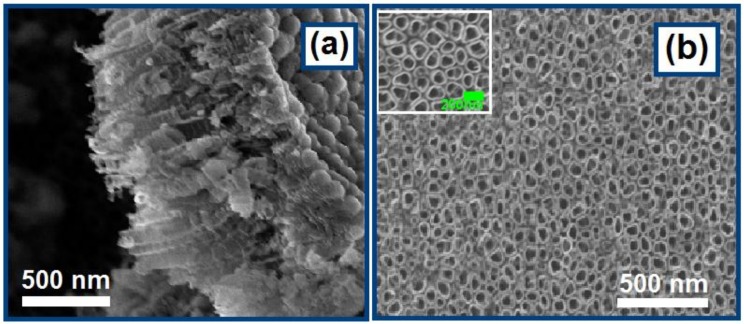
Field-emission scanning electron microscopy (FESEM) images of the titanium dioxide nanotube arrays (TNTAs) prepared by electrochemical anodization of Ti foil: (**a**) cross-section and (**b**) top view.

Figure 1 shows the FESEM images of the titanium dioxide nanotube arrays (TNTAs) prepared by anodization of Ti foil followed by sonication to remove the debris formed on the surface during the anodization. The cross-sectional view of TNTAs in the FESEM images illustrates that the nanotube arrays have an average length of 900 nm. The top view FESEM image of the TNTAs clearly shows the porous nanotube structure, where the diameter of the nanotubes is estimated to be in the range of 90–140 nm (Figure 1b and inset). The rough morphological characteristics of the TNTA show that it is an ideal motif for forming superhydrophobic surfaces.

### 3.2. Surface Functionalization of TNTAs Using ODPA and PFDPA

**Figure 2:**
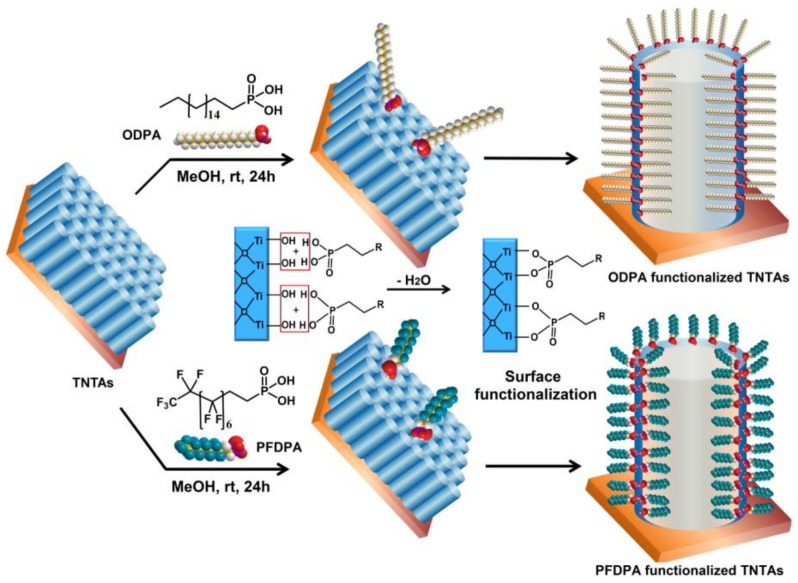
Schematic illustration of the TiO_2_ nanotube arrays (TNTAs) and the conformal surface functionalization of TNTAs with octadecylphosphonic acid (ODPA) or 1H, 1H′, 2H, 2H′-perfluorodecyl phosphonic acid (PFDPA) molecules to form superhydrophobic TNTAs.

Figure 2 presents the schematic illustration of the surface functionalization of TNTAs by simple immersion of the TNTA in ODPA and PFDPA methanol solutions and equilibration of the solution to complete the reaction between the phosphonic acid headgroup in ODPA and PFDPA, and hydroxyl groups on the surface of TNTA, leading to the formation of an ordered, densely packed self-assembled monolayer (SAM). The reaction leads to the formation of strong Ti-O-P bonds exposing the non-polar hydrophobic alkyl and perfluoralkyl moieties towards the ambient. The infrared spectra of the ODPA and PFDPA functionalized surfaces, ODPA@TNTAs and PFDPA@TNTAs, confirm the formation of SAM (see Section 3.4 below). The exposed alkyl or perfluoralkyl moieties ensure the TNTA surface has very low surface energy and is hence superhydrophobic. The polar phosphonic acid end groups in ODPA and PFDPA form strong covalent bonds with surface –OH groups of TiO_2_, resulting in the attachment of the molecules to the TiO_2_ surface via phosphonate ester linkage [48,49]. This process exploits the well-established affinity of the phosphonic acid functional group to the metal oxide surfaces [22]. Metal oxide nanostructures such as TNTAs are ideal substrates for the construction of superhydrophobic coatings, since the trapped air in the grooves of the nanostructures minimizes the water-substrate contact area of the water droplets, resulting in a higher static CA [39,50,51] as expected from the Cassie-Baxter model [52] compared to the SAM modified smooth surface.

### 3.3. Contact Angle and Contact Angle Hysteresis

**Figure 3:**
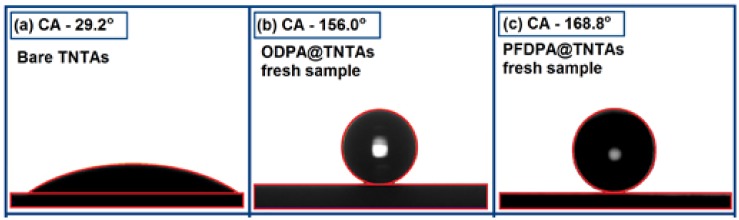
Contact angles of bare TNTA and after functionalization with self-assembled monolayers (SAMs): (**a**) bare TNTAs (**b**) freshly prepared ODPA@TNTAs, and (**c**) freshly prepared PFDPA@TNTAs.

Contact angle (CA) is an important parameter that defines the wettability of a solid surface and has a unique value at a given temperature and pressure for a solid surface in equilibrium with a liquid and vapor. Figure 3 shows a water droplet placed on the bare TNTA prepared by anodization of Ti foil after the sample has been washed with water and sonicated to remove the debris formed in the anodization. As the bare TNTA has free OH groups on the surface, the water droplet spreads and has a low CA of 29.2° ± 1.5° corresponding to a hydrophilic surface. The covalently linked long alkyl or perfluoroalkyl chain interacts to form a high-density packed SAM with low surface energy and superhydrophobic surface, as indicated by the shape of the droplets with contact angle of 156.0° ± 1.5° and 168.8° ± 1.5° for ODPA@TNTAs and PFDPA@TNTAs, respectively. 

**Figure 4:**
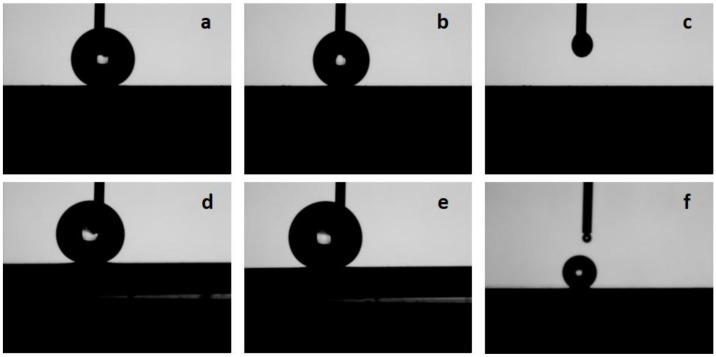
Typical snapshots of the water droplets obtained in the determination of advancing and receding contact angle (CA): (**a**) and (**d**) expanding droplets, (**b**) and (**e**) contracting droplets, and (**c**) and (**d**) wetting as water recedes into the syringe for (**a**–**c**) PFDPA@TNTA and (**d**–**f**) ODPA@TNTA samples.

Another important property of an extremely hydrophobic surface is the stickiness of the water droplets to the surface, which can either be non-adhesive, as in the case of the “lotus state”, or highly adhesive, as in the case of “rose petal state”. A parameter that characterizes this property is the contact angle hysteresis. Contact angle hysteresis is the difference in the CA between the advancing and receding CAs. Figure 4 shows typical examples of the shapes of the water droplets obtained during expansion and contraction of the volume, as water is pumped in and pumped out of the droplet at a rate of 0.4 µL/s. In the case of the PFDPA@TNTA system, the experimentally determined advancing and receding CAs of 155.7° ± 1.5° and 154.9° ± 1.5° yield a CA hysteresis of 0.8^o^, which is within the range of experimental error indicating little or no hysteresis for PFDPA@TNTA. Further details on the measurement of the CA hysteresis are presented in the supplementary information Figure S1. Similar measurements for the ODPA@TNTA system yielded advancing and receding CAs of 153.4° ± 1.5° and 150.4° ± 1.5° and a CA hysteresis of 3.0°. The difference in the CA hysteresis between PFDPA@TNTA and ODPA@TNTA is small, yet it is interesting to note that the increased adhesiveness of the ODPA@TNTA sample as displayed by the retention of a small amount of water droplet on the surface of the ODPA@TNTA as water is pumped back into the syringe, as shown in Figure 4f. In contrast, no water droplet is retained on the surface of PFDPA@TNTA sample, as water is pumped in back into the syringe, as shown in Figure 4c. These results indicate that the superhydrophobicity of the PFDPA@TNTA system concur with the Cassie-Baxter model; on the other hand, the wetting properties of the ODPA@TNTA system are indicative of an intermediate state with a mild transition toward the Wenzel model.

### 3.4. Resilience of the SAM Functionalized TNTA

To determine the resilience of ODPA@TNTAs and PFDPA@TNTAs under harsh conditions, they have been subjected to ultrasonic cavitation, water jet treatment, and abrasion. Several techniques such as CA, DRIFTS, and FESEM were used to characterize the effects of these treatments. 

For ultrasonic resilience test, a high-power horn-sonicator operated at an output power of 225 W, which produces intense cavitation and agitations due to the elastic vibrations of the medium were used to evaluate the resilience of the as-prepared superhydrophobic surfaces of SAM-functionalized TNTA. For instance, a 10 min ultrasonication of ODPA@TNTAs and PFDPA@TNTAs in water showed minimal effect on their superhydrophobicity, as the changes in the CAs values before (156.0 ± 1.5 and 168.8 ± 1.5) and after (151.2 ± 1.5 and 153.3 ± 1.5) treatment are minimal. Further, few or no changes in the CAs were observed when samples were stored overnight under ambient conditions. The superhydrophobicity of the samples were also observed to be resilient against impact caused by pumping water jet to the sample, as shown in the Supplementary Information Figures S1 and S2. In contrast to the resilience exhibited by the superhydrophobicity originating from the ODPA and PFDPA functionalization of TNTA, the superhydrophobicity of TNTA achieved by the commercially available coating paint (RUST-OLEUM@TNTA) is drastically affected by ultrasonication. As presented in Supplementary Information Figure S3, the superhydrophobic RUST-OLEUM@TNTA with CA of 150° degrades to become hydrophilic with CA of < 10° upon 10 min ultrasonication. The reasons for these differences originate from the fact that the SAM in ODPA@TNTAs and PFDPA@TNTAs are covalently linked to the TNTA, whereas only a weak physical bonding holds the paint to the surface of TNTA in the RUST-OLEUM@TNTA sample.

**Figure 5:**
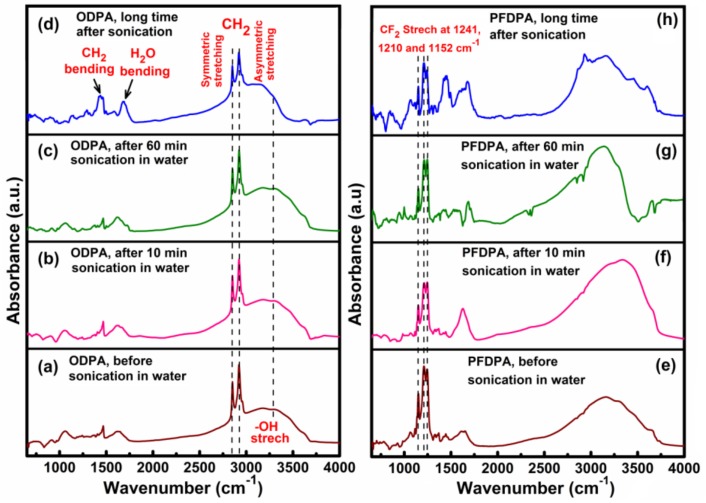
Diffuse reflectance Fourier transform infrared spectroscopy (DRIFTS) spectra of the as-prepared TNTAs functionalized with ODPA and PFDPA and after ultrasonication in water: (**a**–**d**) ODPA@TNTA, and (**e**–**h**) PFDPA@TNTAs; (**a**) and (**e**) as prepared or before ultrasonication, (**b**) and (**f**) after 10 min ultrasonication, (**c**) and (**g**) after additional 60 min ultrasonication, and (**d**) and (**h**) sonicated samples after storing overnight in the ambient.

Infrared spectroscopy was used to unambiguously identify and confirm the presence of ODPA and PFDPA in the functionalized TNTA samples. Figure 5 shows DRIFTS spectra of the as-prepared ODPA@TNTAs and PFDPA@TNTAs, and after the samples have been subjected to utrasonication. The DRIFTS spectra of ODPA@TNTAs exhibit characteristic symmetric and anti-symmetric C–H stretching vibrations at 2850 cm^–1^ and 2919 cm^–1^ due to CH_2_ fragments in the alkyl chain of ODPA (Figure 5a) [53,54]. Moreover, the presence of various CH_2_ bending and C–C stretching modes of alkyl chains was clearly visible, which confirms the successful functionalization of TiO_2_ surface with OPDA [55]. Additionally, a broad peak around 3250 cm^–1^ and a relatively weak peak around 1650 cm^–1^ were attributed to surface adsorbed –OH groups and H_2_O bending vibrations. Interestingly, even after prolonged sonication (10 min followed by 60 min) of the ODPA functionalized nanotubes, the intensity of C–H symmetric and asymmetric stretch remained essentially unaffected, indicating that the strong phosphonate ester linkage (ROPO_2_–) between TiO_2_ and ODPA is unaffected by the vigorous ultrasonication treatment (Figure 5b-d) [56]. Overnight air drying of ODPA@TNTAs samples also showed no detectable change in the C–H stretch modes (Figure 5d). The resilience of the ODPA@TNTAs and PFDPA@TNTAs samples against intense agitation and cavitation is supported by CA measurements before and after 10 min. For the PFDPA@TNTA samples, the DRIFTS spectra exhibited characteristic peaks at 1240 cm^–1^, 1210 cm^–1^, and 1150 cm^–1^ attributed to C–F symmetric and asymmetric stretches of perfluoroalkyl chains, which confirmed presence of the PFDPA molecules on TiO_2_ nanotube surface (Figure 5e–f) [39,57]. Analogous to ODPA@TNTAs, the characteristic –CF_2_ bands of PFDPA@TNTAs samples are unaffected by ultrasonication, as well as storing the sample under ambient conditions.

**Figure 6:**
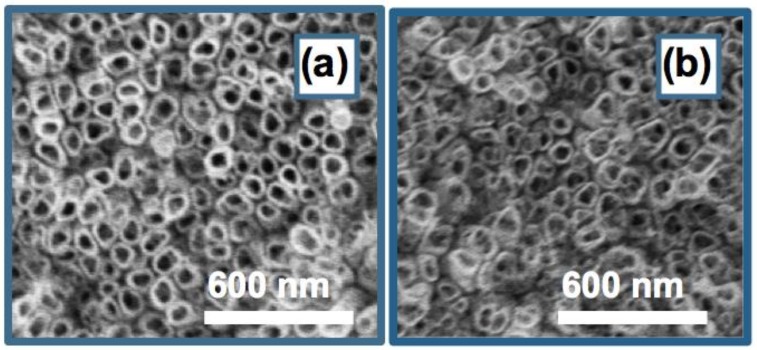
FESEM images of (**a**) ODPA@TNTA and (**b**) PFDPA@TNTA recorded after intense cavitation by ultrasonication in water for 10 min followed by additional 60 min.

The structural and morphological integrity of TiO_2_ nanotubes in TNTAs is critical for maintaining the superhydrophobicity of the ODPA@TNTAs and PFDPA@TNTAs samples. Figure 6 shows the FESEM images of ODPA@TNTAs and PFDPA@TNTAs samples recorded after ultrasonic treatment. Comparison of the FESEM images recorded before (Figure 2) and after ultrasonic treatment showed that these treatments have no adverse effect on the structural and morphological integrity of TiO_2_ nanotube in TNTA. The samples are resilient under these conditions.

**Figure 7:**
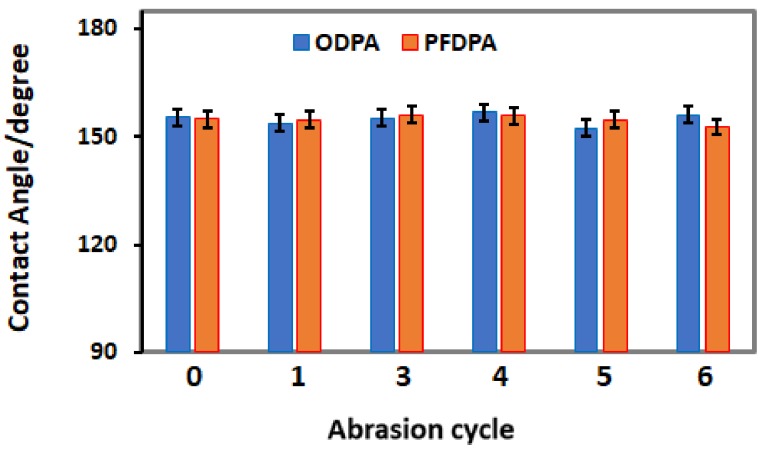
CA versus number of abrasion cycles: ODPA@TNTA (blue bar) and PFDPA@TNTA (orange bar) samples. An abrasion cycle consists of pushing the sample with 50 g weight on it through a 320-grit sand paper for a distance of 5 cm. The CA was measured at the end of each cycle.

The resilience of superhydrophobic properties of ODPA@TNTA and PFDPA@TNTA samples were determined against abrasion, as shown in Figure 7. In the abrasion test, a 50 g weight was placed on the ODPA@TNTA or PFDPA@TNTA sample with the superhydrophobic surface resting on the 320-grit sand paper (see Figure S5 in Supplementary Information). The sample was pushed through the 320-grit sand paper for a distance of 5 cm using a tweezer to cause abrasion. The damage due to abrasion accumulated as the cycle is repeated. The CA was then determined at the end of each cycle and plotted as presented in Figure 7. The abrasion tests show that CA values determined at the end of each cycle lie within the range of experimental error, even after 5 abrasion cycles, thereby indicating the robustness of SAM bonding to the TiO_2_ nanotubes and the structural integrity of the TNTA system. This is similar to Steele et al.’s report of unprecedented mechanical durability exhibited by a nonwetting material following abrasion [58]. The resilience test on the ODPA@TNTA and PFDPA@TNTA samples achieved by applying stressors in the form of ultrasonication, water jet impact, and abrasion and characterized by CA, DRIFT, and SEM showed that applied stress has minimal effect on the properties of the samples, indicating the robustness of the systems.

### 3.5. Corrosion Testing and Electrochemical Analysis

**Figure 8:**
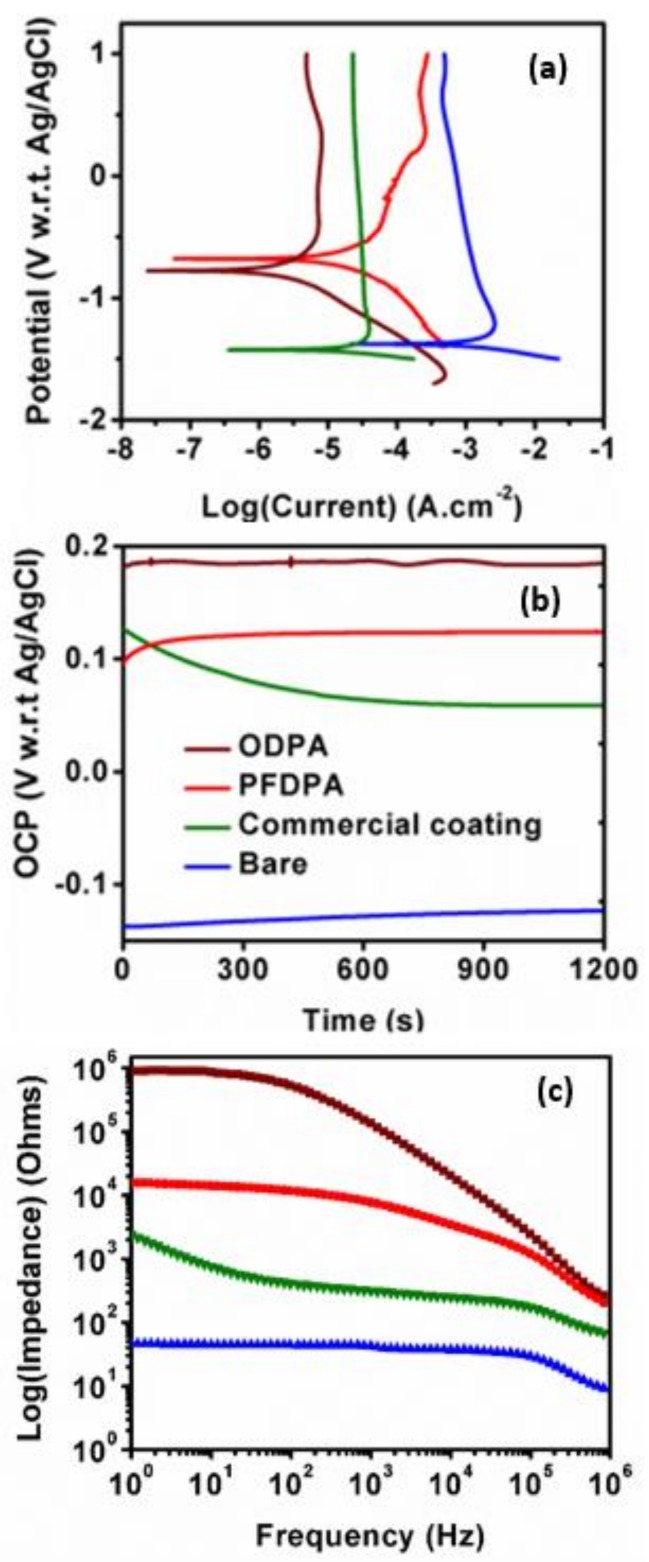
(**a**) Tafel plots, (**b**) plots of open circuit potential (OCP) versus time, and (**c**) Bode plots for the bare TNTA, ODPA@TNTA, PFDPA@TNTA, and RUST-OLEUM@TNTA.

The resistance of the samples to corrosion was performed in 3.5 wt. % NaCl in three-electrode setup. Figure 8a shows the Tafel plots, wherein higher *E*_corr_ values can be observed for ODPA and PFDPA SAMs than for the bare TNTAs and commercial coating incorporated TNTAs. Insulation due to the SAM and coating is reflected in the increased resistance of these samples compared to bare TNTA sample. Evolution of open circuit potential (OCP) with time is shown in Figure 8b, and it reveals similar trend as Tafel curves (in Figure 8a). Polarization resistance (*R*_p_) of the SAMs was calculated using Equation (1) [59], in which *B* is a constant related to Tafel constants (anodic, *β*_a_, and cathodic *β*_c_) by Equation (2). *I*_corr_ is the corrosion current, which is obtained at the intersection of Tafel slopes. Values of *β*_a_, *β*_c_, *E*_corr_, *I*_corr_*,* and *R*_p_ are listed in Table 1. 

The polarization resistance for bare TNTAs, as obtained herein, is very small compared to that of PFDPA and ODPA-coated SAMs (see Supplementary Information Figure S4). Moreover, polarization resistance of ODPA and PFDPA SAMs is higher than that of the RUST-OLEUM@TNTA, which has commercial coating (see Supplementary Information Figure S4). Superior corrosion resistance of ODPA and PFDPA SAMs compared to commercial RUST-OLEUM coatings is therefore evident in saline (NaCl) water solution on the basis of polarization resistance and corrosion currents.

(1) $$\;R_{p}=\frac{B}{I_{corr}}\;$$

(2) $$\;B=\frac{\beta_{a}\beta_{c}}{2.3\left(\beta_{a}+\beta_{c}\right)}\;$$

(3) $$\;\varphi =1-\frac{Z}{Z_{Bare}}\;$$

EIS Bode plots (Figure 8c) show orders of magnitude higher impedance in the low frequency region for ODPA and PFDPA SAMs compared to the RUST-OLEUM coated TNTA. The bare TNTA exhibits the lowest magnitude of impedance, implying that the presence of SAM and commercial coatings (RUST-OLEUM) dramatically increase the capacitive component of the impedance in the low-frequency region of the impedance spectra. Using the impedance magnitudes at the lowest frequency, the approximate surface coverage, *φ* (%), was calculated using Equation (3) [26,60], and listed in Table 1. Equation (3) *Z* is the impedance of the SAMs of the commercial coating, and *Z_Bare_* is the impedance of the bare TNTAs. Close to 100% surface coverage was obtained with ODPA and PFDPA SAMs, while 98% surface coverage was obtained with the commercial coating.

A major conceptual advancement is definitive evidence that the alkylphosphonate monolayer on nanostructured TiO_2_ surfaces neither desorbs nor detaches from the surface when subjected to ultrasonication, water jet impact, and abrasion. The resilience under extreme cavitation was notable for two reasons: First, the nanostructured TiO_2_ being a ceramic was expected to undergo brittle fracture under cavitation, but this did not occur. Second, the commonly used SAM based on headgroups such as alkanethiols on noble metal surfaces and alkane hydroxamic acids on metal and metal oxide surfaces is known to undergo slow desorption in aqueous solutions and oxidation in the presence of air [61,62,63]. While phosphonate monolayers are recognized to be robust [64,65], their resilience to corrosion, ultrasonication, water jet impact, and abrasion has not been reported to the best of our knowledge. The significance of this work stems from the fact that OPDA and PFDPA-functionalized TNTA, achieved using a simple immersion technique presented in this study, have excellent anti-wetting surface properties, in addition to being resilient against applied stressors such as corrosion in saline water, ultrasonication, water jet impact, and abrasion. Further, nanostructured TiO_2_ overlayers can be easily formed on technologically important substrates such as stainless steel, glass, quartz, silicon, and plastic [66,67,68,69,70,71,72].

## 4. Conclusions

Robust and resilient superhydrophobic materials have been fabricated from TiO_2_ nanotube arrays by anodization of Ti foil and subsequent functionalization with ODPA and PFDPA. The superhydrophobic PFDPA@TNTA exhibits virtually no CA hysteresis, whereas ODPA@TNTA has a small CA hysteresis of 3° and consequently was observed to have a stronger affinity to adhere to small water droplets. Both these materials, PFDPA@TNTA and ODPA@TNTA, were observed to be very resilient against intense cavitation upon ultrasonication, water-jet impact, and abrasion. No structural damage to TiO_2_ nanotubes or delamination of the SAM layer were observed when these materials were subjected to cavitation upon ultrasonication, water-jet impact, and abrasion. In addition, PFDPA@TNTA and ODPA@TNTA exhibited enhanced resistance to corrosion due to the protective coating of the SAM layer compared to uncoated bare TNTA. These findings increase the possibility of developing practical applications based on surface-functionalized TNTAs. 

## Figures and Tables

**Table 1:** Corrosion performance data for the octadecylphosphonic acid (ODPA) and 1H, 1H′, 2H, 2H′-perfluorodecyl phosphonic acid (PFDPA) self-assembled monolayers (SAMs), and commercial coating incorporated TiO_2_ nanotube arrays (TNTAs).

	*β*_a_ (*V.decade*^−1^)	*β*_c_ (*V.decade*^−1^)	*E*_corr_ (V w.r.t. Ag/AgCl)	*I*_corr_ (µA.cm^−2^)	*R*_p_ (Ω.cm^−2^)	*φ* (%)
ODPA SAM	0.250	−0.178	−0.774	1.48	181676	99.99
PFDPA SAM	0.329	−0.153	−0.677	5.75	21626	99.72
Commercial coating	0.079	−0.052	−1.452	10.91	6036	98.16
Bare	0.481	−0.099	−0.774	729.45	74	0

## Supplementary Materials

The following are available online at http://www.mdpi.com/2079-4991/8/10/783/s1; Figure S1: The water repelling tendency of the ODPA functionalized TNAs surface before ultrasonic wave treatment; Figure S2: Resilience of the superhydrophobic character of an ODPA coated TNTA surface following long time exposure to ambient environment after ultrasonic treatment; Figure S3: (**a**) Contact angles of commercial coating incorporated TiO_2_ nanotube arrays. (**a**) Before ultrasonication; (**b**) and (**c**) After ultrasonication in water for 10 and 60 min, respectively. (**d**) After ultrasonication in methanol for 10 min; Figure S4: Tafel plots showing the values Tafel constants, Ecorr and Icorr for (**a**) ODPA SAM (**b**) PFDPA SAM (**c**) commercial coating and (**d**) bare TiO_2_ nanotubes; Figure S5: Photograph of abrasion test in process. A 50 g weight is placed on the SAMcoated TNTA sample and then the sample is pushed across a 320-grit sand paper for a distance of 5 cm using a pair of (blue colored) tweezers, Video S1: ODPA-coated TNTA_advancing contact angle measurement; Video S2: ODPA-coated TNTA_receding contact angle measurement; Video S3: PFDPA-coated TNTA_advancing contact angle measurement; Video S4: PFDPA-coated TNTA_receding contact angle measurement; Video S5: SAM-coated TNTA Abrasion Test.
