# Supplementary material for: Resistance of Superhydrophobic Surface-Functionalized TiO2 Nanotubes to Corrosion and Intense Cavitation
Source: Nanomaterials (Basel). 2018 Oct 2;8(10):783. doi: 10.3390/nano8100783 (PMC6215275; doi:10.3390/nano8100783)
Supplement: Supplementary file 1 [file nanomaterials-08-00783-s001.zip › Revised ESI_Nanomaterials.pdf]

Supplementary Information

Resistance of Superhydrophobic Surface-Functionalized TiO_2 Nanotubes to Corrosion and Intense Cavitation

Weidi Hua[1†], Piyush Kar[1†], Partha Roy[2], Lintong Bu,[1] Lian C.T. Shoute,[1] Pawan Kumar[1] and Karthik Shankar*[1]

[1]*Department of Electrical and Computer Engineering, University of Alberta, 9211-116 St, Edmonton, Alberta T6G 1H9, Canada*

[2]*Department of Chemistry, Central University of Rajasthan, NH-8, Bandar Sindri, Rajasthan 305817, India*

†Authors who contributed equally.

*Email: kshankar@ualberta.ca

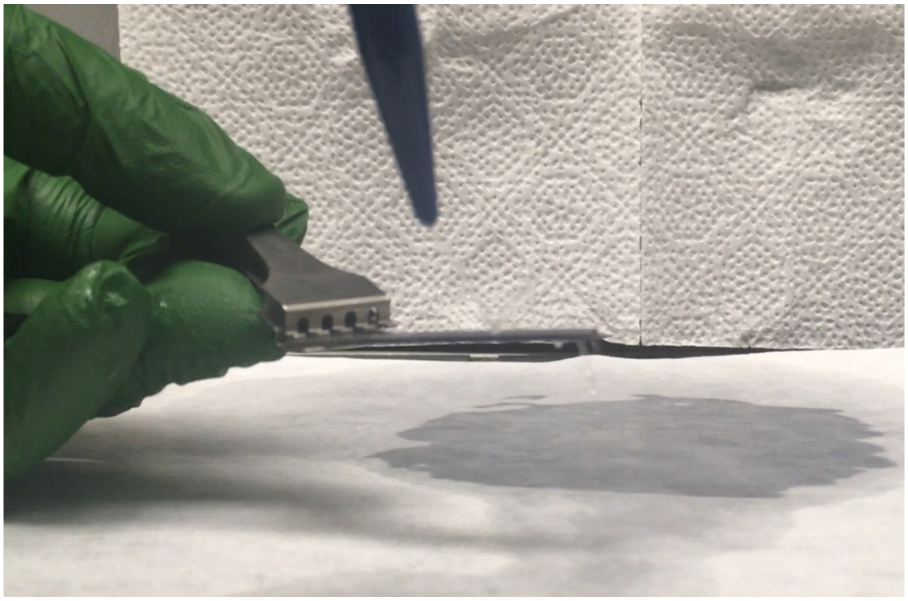

Figure S1. The water repelling tendency of the ODPA functionalized TNAs surface before ultrasonic wave treatment.

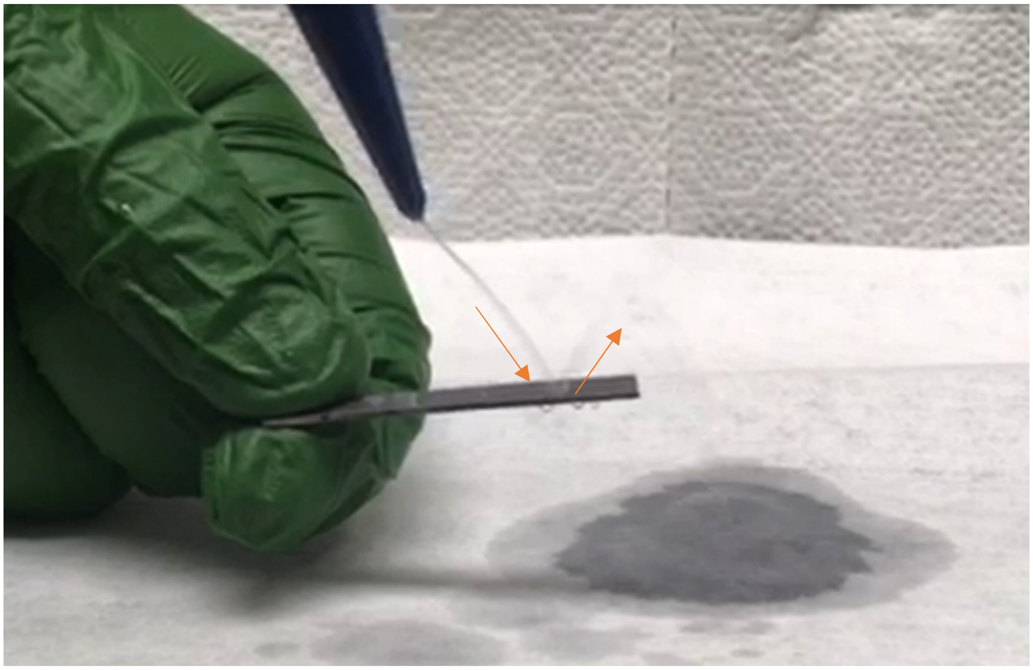

Figure S2. Resilience of the superhydrophobic character of an ODPA coated TNTA surface following long time exposure to ambient environment after ultrasonic treatment.

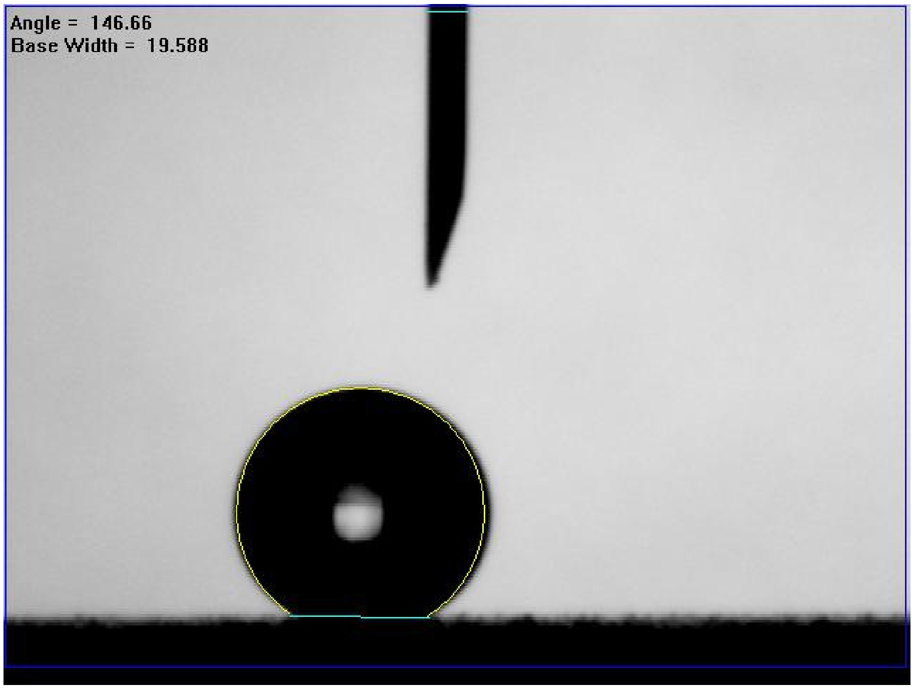

(a)

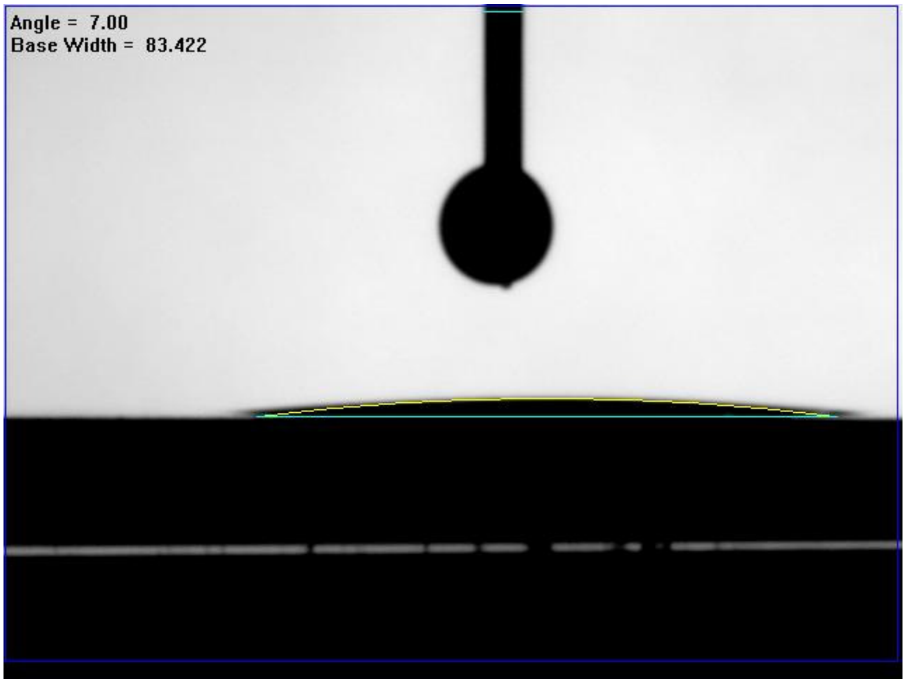

(b)

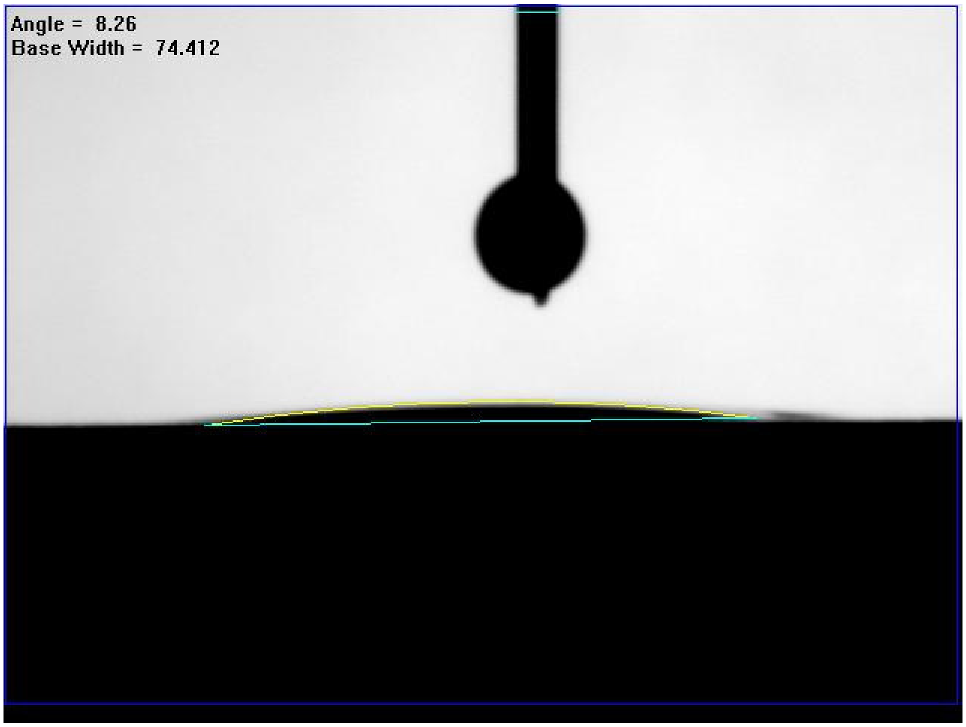

(c)

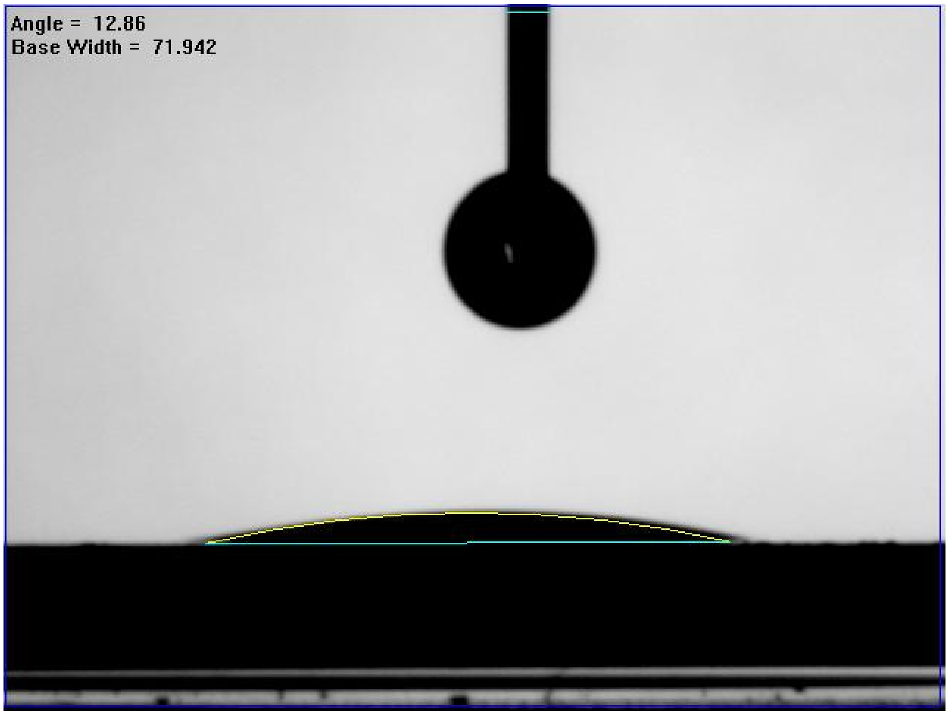

(d)

Figure S3. (a) Contact angles of commercial coating incorporated TiO_2 nanotube arrays. (a) Before ultrasonication; (b) and (c) After ultrasonication in water for 10 and 60 minutes, respectively. (d) After ultrasonication in methanol for 10 minutes.

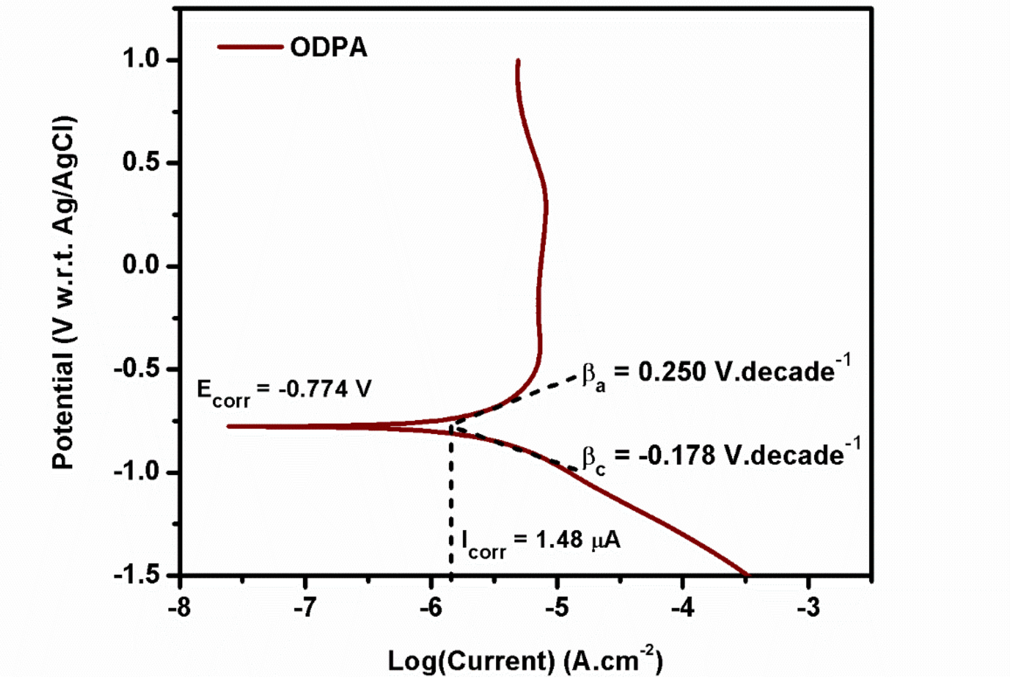

(a)

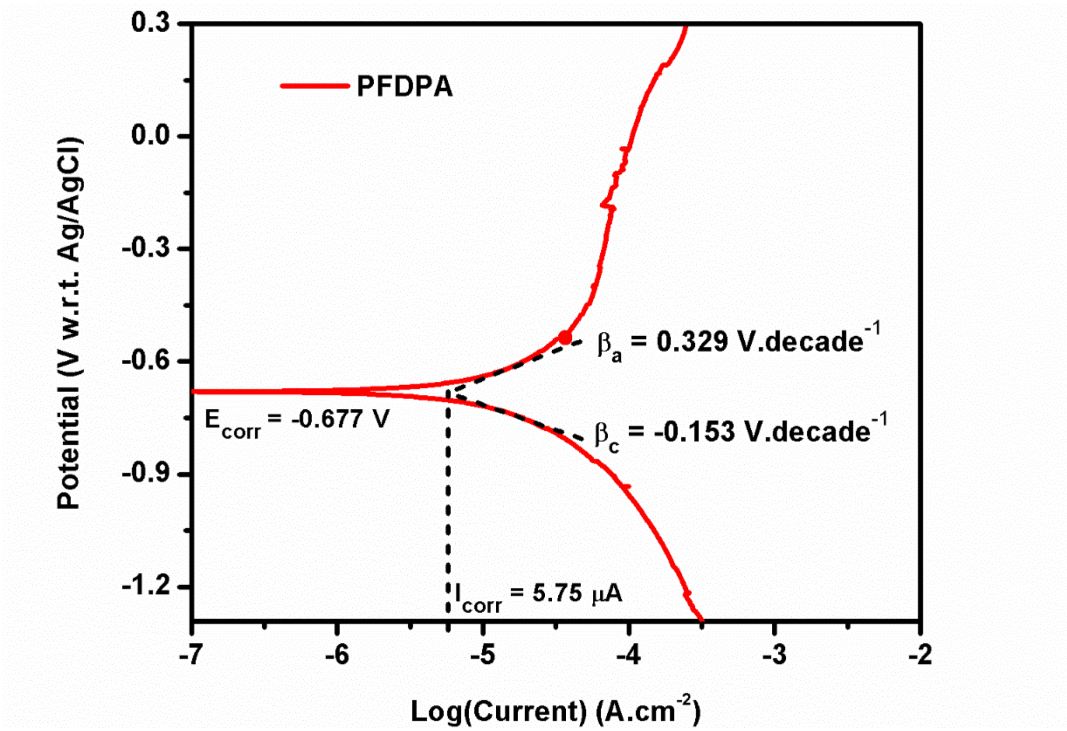
PFDPA
βa = 0.329 V.decade-1
βc = -0.153 V.decade-1
Ecorr = -0.677 V
Icorr = 5.75 μA
Potential (V w.r.t. Ag/AgCl)
Log(Current) (A.cm-2)
0.3
0.0
-0.3
-0.6
-0.9
-1.2
-7
-6
-5
-4
-3
-2

(b)

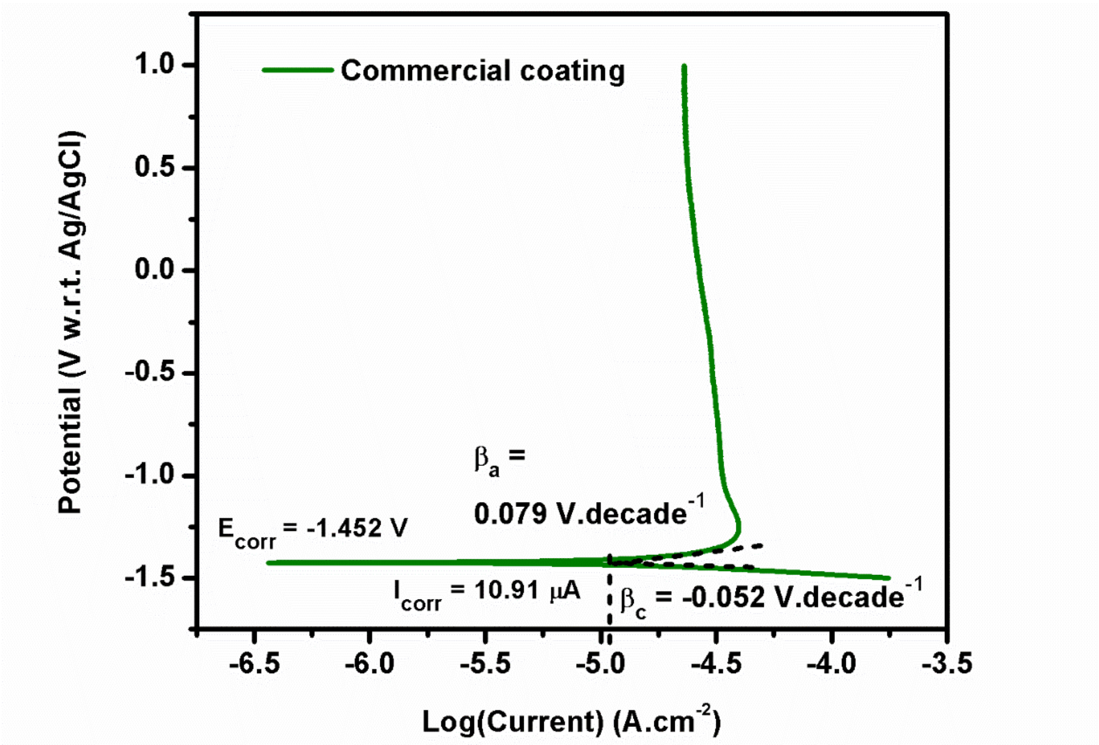
Commercial coating
βa =
0.079 V.decade-1
Ecorr = -1.452 V
Icorr = 10.91 μA
βc = -0.052 V.decade-1
Potential (V w.r.t. Ag/AgCl)
Log(Current) (A.cm-2)
1.0
0.5
0.0
-0.5
-1.0
-1.5
-6.5
-6.0
-5.5
-5.0
-4.5
-4.0
-3.5

(c)

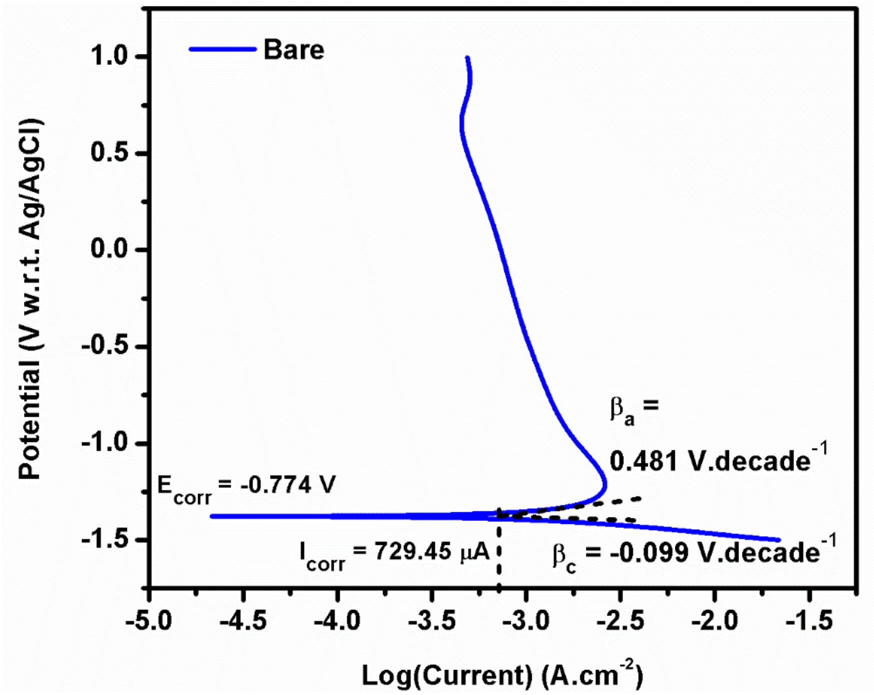

(d)

Figure S4. Tafel plots showing the values Tafel constants, Ecorr and Icorr for (a) ODPA SAM (b) PFDPA SAM (c) commercial coating and (d) bare TiO_2 nanotubes.

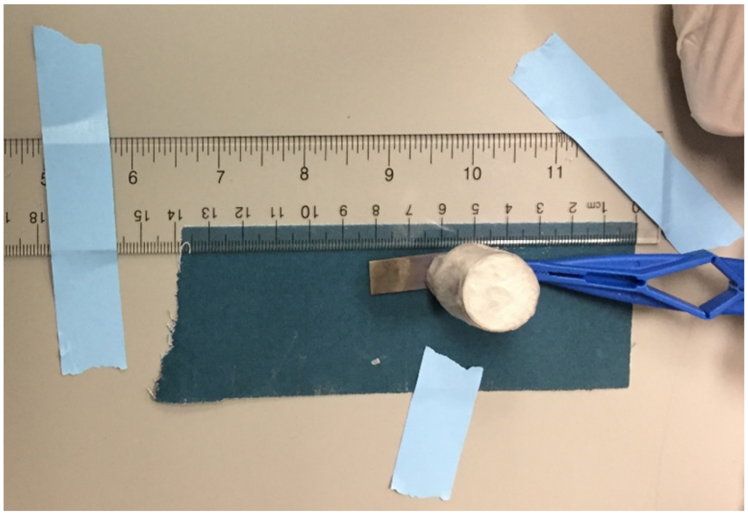

Figure S5. Photograph of abrasion test in process. A 50 gram weight is placed on the SAM-coated TNTA sample and then the sample is pushed across a 320-grit sand paper for a distance of 5 cm using a pair of (blue colored) tweezers.
